# Carbon Kuznets curve in China: Nighttime light analysis in prefecture-level cities

**DOI:** 10.1016/j.heliyon.2024.e36312

**Published:** 2024-08-14

**Authors:** Xiaoqi Zheng, Jiaying Wang, Xiangbo Xu, Ran Yu, Sheng Zhang

**Affiliations:** a School of Economics, Nanjing University of Posts and Telecommunications, No.9, Wenyuan Road, Nanjing, 210023, China; b Institute of Geographical Science and Resources, Chinese Academy of Sciences, 11A, Datun Road, Chaoyang District, Beijing, 100101, China; c School of Environment, Renmin University of China, No. 59 Zhongguancun Street, Haidian District Beijing, 100872, China; d School of Environment, Tsinghua University, No. 30 Shuangqing Road, Haidian District Beijing, 100084, China

**Keywords:** CO_2_ Kuznets curve, Nighttime light brightness, Prefecture-level city, China

## Abstract

Analysing the relationship between economic growth and carbon emissions may help China to determine how to reduce carbon emissions. This study examined 221 Chinese prefecture-level cities using data from 2003 to 2018 to investigate the possible inverted U-shaped relationship between carbon emissions and economic growth, based on per capita nighttime light brightness. This revealed a significant inverted U-shaped relationship between per capita nighttime and carbon emissions with a per capita GDP of 139.8 thousand Yuan corresponding to the turning point. For China to achieve its carbon emissions peak before 2030, the per capita GDP growth rate would need to be at least 6.6 %. Therefore, for help prefecture-level cities to achieve peak carbon, they must strive towards high-quality economic growth and accelerate transformation of economic growth modes to improve low-carbon pilot policies, and such projects should be implemented in more cities. Efforts to achieve peak should be accelerated first in eastern cities and later in western cities.

## Highlights

• Inverted U-shaped curve relating economic growth and carbon emissions revealed.
• Cities that implement low-carbon pilot policies may reach peak carbon earlier.
• In China, eastern cities will likely reach peak carbon earlier than western cities.

## 1 Introduction

The sixth Intergovernmental Panel on Climate Change released assessment report, titled the “*Climate Change 2022: Mitigation of Climate Changes*”, emphasises that limiting global warming to 1.5 °C requires all countries to increase the rate and intensity of greenhouse gas emission reductions [1]. In recent years, the Chinese government has taken a proactive stance towards climate change. In 2020, China announced its carbon peak and carbon neutrality targets, garnering significant attention worldwide. Energy-related carbon emissions already account for approximately 28 % of the global emissions total [2]. The emergence of a global carbon peak and future reduction paths depend largely on China's carbon emission reduction trajectory. Consequently, the timing of China's overall carbon emissions peak hinges on the timing of the peak in each region, particularly in prefecture level cities. By the start of 2023, more than 20 provinces, along with some prefecture-level cities, had released implementation plans for their regional carbon peak. Chinese-style modernisation, first proposed at the 20th National Congress of the Communist Party of China, requires carbon emissions to peak and then decline steadily with a continuous increase in economic development; therefore, China and all regions must explore the balance between economic growth and green development. Consequently, further analysis of the relationship between economic growth and carbon emissions is essential to achieve peak carbon in China peak as soon as possible. Recent studies on China's carbon emission peak have focused on policy assessment, energy model predictions, and technological innovation [[3], [4], [5], [6]]. The relationship between economic growth and carbon emissions has been studied [7,8], with the most representative analysis that on the CO_2_ Environmental Kuznets Curve (CKC) hypothesis derived from Environmental Kuznets Curve hypothesis. The CKC reveals the complex relationship between economic growth and environmental protection. When used to analyse China's carbon emissions, the Kuznets curve can provide scientific reference data and guidance for policymakers; further, it can be used to promote the virtuous cycle of economic development and environmental protection and to achieve the dual goals of sustainable development and carbon emission reduction.

Kuznets first studied the relationship between income inequality and economic development, finding that they exhibited an inverted U-shaped relationship [9]. This was later extended by Grossman, who developed the environmental Kuznets hypothesis to describe the relationship between economic growth and environmental pollution [10]. According to the CKC, environmental quality may gradually deteriorate with an increase in the economic development during the early stages of economic growth. However, beyond a certain threshold of economic development, environmental quality gradually improves with further economic growth. Recent studies on the CKC's inverted U-shaped relationship have generated conflicting results owing to differences in data sources or methods. Such relationship has been reported between carbon emissions and GDP [[11], [12], [13], [14]] and between per capita emissions and per capita GDP [[15], [16], [17]]. However, various relationship between carbon emissions and economic growth has been reported, including linear, positive U-shaped, N-shaped, and even inverted N-shaped [[18], [19], [20], [21], [22]], and the inverted U-shaped environmental Kuznets curve has been critically questioned [23]. Differences in sample selection, core explanatory variables, control variables and measurement methods can all lead to the loss of the environmental Kuznets curve [24]. CKC studies exhibit several limitations. First, most Kuznets curve studies utilise provincial panel data, with few using prefecture level city data, they typically use per capita GDP as the core explanatory variable, and don't consider innovation. Second, few Kuznets curve studies have examined carbon peak policie. Consequently, and considering that nighttime brightness in China is an indicator of economic development [25], particularly in prefecture-level cities [26], nighttime light remote sensing data should be used as the core explanatory variable to verify the inverted U-shaped relationship between economic growth and prefecture level city carbon emissions in China. Kuang et al. showed that nighttime light brightness is highly correlated with economic growth [27], and Zhang et al. used nighttime light data to invert carbon emissions [28]. Studies that have identified an inverted U-shaped relationship between economic growth and carbon emissions have also analysed the position of the inflection point.

This study examines the relationship between carbon emissions and economic growth from 2003 to 2018, based on Chinese prefecture-level city nighttime light data. Prefecture-level city data exhibits an internal structure that the provincial data cannot reflect, providing more stable results. The nighttime light data used here is both novel and reliable. Second, this study verifies the inverted U-shaped curve at the city level in China, finding that achieving peak carbon by 2030 will require average annual per capita GDP growth rate at least 6.6 %. This provides a scientific reference for formulating carbon-emission reduction policies. Third, a heterogeneity analysis is conducted from the perspective of policy changes, regional economic differences, and city size, and the Kuznets curve of China's carbon emissions is studied in more detail than in previous studies. The remainder of this paper is structured as follows. Section 2 introduces the methods, Section 3 presents the empirical analysis results, Section 4 discusses the results, and Section 5 offers concluding remarks and recommendations based on the findings.

## 2 Methods

### 2.1 Model setup

The traditional Environmental Kuznets Curve model considers only the correlation between economic growth and environmental pollution, disregarding crucial components such as industrial structure and technological advancement [29]. This study highlights the correlation between economic growth and carbon emissions while exploring their quadratic relationship. Control variables such as population density, the capital–labour ratio, the industrial elevation, the urbanisation rate and annual electricity consumption are considered to avoid omitting important variables. Thus, the following model was established.

(1) $${cityemi}_{it}=\alpha_{0}+\beta_{1}{nightlcap}_{it}+\beta_{2}{{nightlcap}_{it}}^{2}+\lambda_{m}{Control}_{mit}+\delta_{i}+\theta_{t}+\varepsilon_{it}$$

where, ${cityemi}_{it}$ is the CO_2_ emissions level of prefecture-level city *i* in year *t*; ${nightlcap}_{it}$ is the per capita nighttime light brightness of city *i* in year *t*, used to indicate the level of economic growth; ${{nightlcap}_{it}}^{2}$ is the quadratic term of per capita nighttime light brightness; ${Control}_{mit}$ represents the control variables *lpopden*, *lcaplar*, *indupd*, *urban and ltotele*. The variables *lpopden*, *lcaplar*, *indupd*, *urban* and *ltotele* refer to the logarithm of population density, logarithm of capital–labour ratio, upgrading of industrial structure, urbanisation rate and logarithm of annual electricity consumption, respectively; $\beta_{1}$ and $\beta_{2}$ represent the coefficients of ${nightlcap}_{it}$ and ${{nightlcap}_{it}}^{2}$ respectively. $\lambda_{m}$ represents the to-be-estimated parameters of five control variables; $\alpha_{0}$ is the intercept term, $\delta_{i}$ is the city fixed effect, $\theta_{t}$ is the time fixed effect, and $\varepsilon_{it}$ is a random disturbance term. Several relationships between carbon emissions and economic growth exist.

①When $\beta_{1}$ = 0, $\beta_{2}$ ≠ 0, cityemi and nightlcap exhibit a linear relationship.
②When $\beta_{1}$ > 0, $\beta_{2}$ < 0, cityemi and nightlcap exhibit an inverted U-shaped curve relationship.
③When $\beta_{1}$ < 0, $\beta_{2\;}$ > 0, cityemi and nightlcap exhibit a positive U-shaped relationship.
④When ${\;\beta }_{1}$ = 0, $\beta_{2}$ = 0, it indicates that carbon emissions are not affected by the economic level.

### 2.2 Variables

#### 2.2.1 Response variable

The variable cityemi refers to levels of CO_2_, the most important greenhouse gas in China, which is responsible for approximately 84 % of the country's total greenhouse gas emissions. With the continued increase in China's urbanisation rate, prefecture-level cities are becoming centres of economic activity, increasing carbon emissions. Urban areas in China contribute up to 85 % of the country's overall emissions [30]. Consequently, the level of carbon emissions from prefecture-level cities were used as considered as the response variable.

#### 2.2.2 Core explanatory variable

The variable nightlcap refers to the nighttime light brightness, which provides a good indication of, and is highly correlated with economic development. As few studies have used nighttime light brightness to explore the relationship between economic growth and carbon emissions. This study used corrected annual nighttime light brightness data for Chinese prefecture-level cities from 2003 to 2018 and retrieved from Wu et al. [31]. By combining this data with household population data, the per capita nighttime light brightness variable was obtained.

#### 2.2.3 Control variables

The variable popden was calculated as the ratio of the total population at the end of the year to the urban area. The upward trend in population density is a clear indication of urbanisation. As population density grows in cities there is an increase in consumer demand, production expansion, and a subsequent increase in resource and energy consumption. However, cities with high population densities usually achieve higher levels of economic development, and the concentration of both population and industry facilitates the diffusion of knowledge and technology, promoting efficient resource consumption and reducing CO_2_ emissions.

The variable caplar, the ratio of fixed asset investments to the number of employees, to some extent reflects technological development level. Higher caplar values tend to be associated with capital-intensive industries, which often have higher technological levels [32]. Higher technological levels typically imply greater energy efficiency and lower pollutant emissions. Consequently, controlling caplar can help to reduce CO_2_ emissions.

The variable indupd, the ratio of the output value of tertiary industry to that of secondary industry, reflects the upgrading of the industrial structure and thus the level of advanced industrial development. According to Chenery's industrialisation stage theory, industrial structures evolve from low to high-level structures, with the and dominant industries progressing sequentially from agriculture to industry and then to the tertiary sector.

The variable *urban* refers to the urbanisation rate, expressed as the ratio of the urban population to the total population. The Urbanisation rate is an important indicator of the economic and social development of a country or region. An increase of urbanisation rate translates to more employment opportunities and improvements in living conditions; however, it may also lead to environmental pollution and resource consumption.

The variable *ltotele* refers to annual electricity consumption. Electricity is an important energy source for modern economic activity; change in this variable reflect fluctuations in economic activity. Including these variables help to achieve a more accurate evaluation of the effects of the core explanatory variable on the response variable in this research.

### 2.3 Data source

The Chinese government has not yet released any prefecture-level city carbon emissions data. This study therefore adopted the CO_2_ emission data of China's prefecture-level cities, from Shan et al. [[33], [34], [35], [36]]. The carbon emissions calculated by Shan et al. include those resulting from both energy-related activities and industrial production process. The data were downloaded from the CEADs database (https://www.ceads.net/), which is subject to data integrity issues, with some cities having incomplete records and significant data gaps before 2003. To ensure balanced panel-data analysis, the study selected data for 221 prefecture-level cities in China with better data quality from 2003 to 2018. Economic data for the prefecture-level cities, including the total registered population, population density, GDP, added value of secondary industries, and fixed asset investments, were sourced from the *China City Statistical Yearbook* for each year. When data were not available, the arithmetic mean of adjacent years was used. Nighttime light brightness data were obtained from Wu et al. [31]. Table 1 presents the descriptive statistical analysis of the variables.

**Table 1:** Descriptive statistics analysis of the main variables.

Variable	N	mean	sd	min	max
*cityemi*	3536	36.31	37.68	0.949	436.2
*nightlcap*	3536	23.50	47.03	0.412	522.8
*lpopden*	3536	5.863	0.865	2.296	7.882
*lcaplar*	3536	12.05	0.861	8.901	13.98
*indupd*	3536	0.867	0.431	0.129	4.347
*urban*	3536	0.503	0.173	0.112	1.00
*ltotele*	3535	13.01	1.173	9.241	16.61

To determine whether the model had significant multicollinearity problems, the correlation coefficients among the main variables were calculated. The results are presented in Table S1. Most of the correlation coefficients are less than 0.3, indicating that there is no significant multicollinearity among the variables. Additionally, in practical econometric analysis, the logarithms of the three parameters (population density, capital–labour ratio, and annual electricity consumption) were taken separately to reduce the interference of heteroscedasticity.

## 3 Results

### 3.1 Benchmark regression analysis

Pre-diagnostic tests were conducted for each variable by applying unit root and co-integration tests. Based on the results of the unit root test (Table S2), the null hypothesis was rejected, indicating that there was no unit root and that the sequence was stationary. The co-integration test results (Table S3) reveals that long-term co-integration between all the variables. The Hausman test was used to determine whether a fixed effects or random effects model was appropriate: the fixed effects model was identified as more suitable (Table S4). Thus, a two-way fixed-effects model was used for benchmark regression analysis. Owing to space limitations, Tables S2, S3 and S4 are provided in the Supplementary Materials.

Based on the benchmark regression results presented in Table 2, there was a distinctive inverted U-shaped relationship between CO_2_ emissions and nighttime light brightness (based on in Model 1). This finding remained valid even when the control variables were considered (Model 2). Thus, carbon emissions at the city level tend to increase as economic development improves, whereas they begin to decline once the level of economic development reaches a certain threshold. This finding is consistent with CKC theory. The relationship between the control variables (*lpopden*, *lcaplar*, *indupd*, *urban*, and *ltotele*) and carbon emissions is consistent with expectations, albeit with low significance or without significance. Population density was negatively correlated with carbon emissions. As population density increases, there is a corresponding concentration of resource elements, leading to greater efficiency in resource utilisation. Such concentration often fuels technological advancements, which positively affects carbon reduction efforts, ultimately leading to a concentration effect that surpasses the scale effect [37]. The *lcaplar* coefficient was negative and statistically significant at the 5 % level. As a representation of technological development, higher ratios indicates more advanced technical levels. Advanced production technologies have been shown to significantly reduce carbon emissions, highlighting the importance of a high capital–labour ratio in a sustainable economy. Although the degree of the impact is not significant, upgrading of industrial structure negatively affects carbon emissions. Pollution and emissions can be reduced by optimizing and upgrading the industrial structure and accelerating tertiary industrial development.

**Table 2:** Results of benchmark regression analysis.

Variable	*cityemi*	
	Model 1	Model 2
*nightlcap*	0.277**	0.262**
	(2.52)	(2.32)
*nightlcap*^*2*^	−0.0005**	−0.000472**
	(−2.47)	(−2.51)
*lpopden*		−4.926
		(−0.39)
*lcaplar*		−5.030**
		(−2.19)
*indupd*		−0.126
		(−0.04)
*urban*		15.910*
		(1.67)
*ltotele*		9.737*
		(1.73)
Constant	16.831***	−25.273
	(7.43)	(−0.29)
Time-fixed effect	Yes	Yes
City-fixed effect	Yes	Yes
Observations	3536	3535
Number of id	221	221
R-squared	0.234	0.274
Utest	Yes	Yes

Note: ***, **, and * represent significance at the level of 1 %, 5 %, and 10 %, respectively. t-statistics are shown in parentheses.

These findings support the CKC hypothesis; however, according to Lind and Mehlum, when the real relationship is convex and monotonous, the model estimation wrongly produces an extreme point and inverted U-shaped relationship [38]. Thus, a post-diagnostic test was conducted to check the reliability of the inverted U-shaped relationship in Models 1 and 2 using the ‘Utest’ test in Stata. Both Models 1 and 2 passed the test (Table 2).

**Fig. 1:**
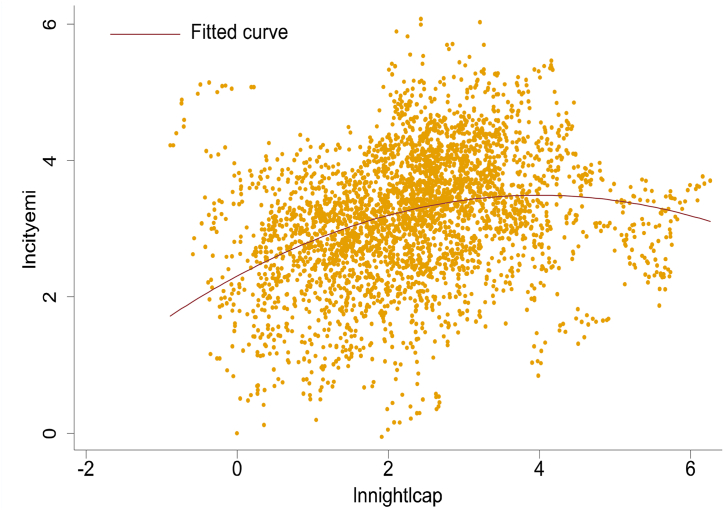
Scatter plot and fitting curve of *lncityemi* and *lnnightlcap*.

A scatter plot and fitted curve of the logarithms of carbon emissions (*lncityemi*) and per capita nighttime light brightness (*lnnightlcap*) are shown in Fig. 1. An inverted U-shaped relationship was observed between *lncityemi* and *lnnightlcap*, consistent with the benchmark regression analysis results. Linear regression was applied to identify the corresponding relationship between per capita nighttime light brightness and per capita GDP (Table S5). A per capita GDP of 139800 Yuan (2018 constant price), was estimated to correspond to the inflection point of per capita nighttime light brightness. China's per capita GDP in 2023 was 89400 Yuan. For China to achieve peak carbon before than 2030, the per capita GDP growth rate needs to be at least 6.6 %.

### 3.2 Robustness test

To ensure the credibility of the benchmark regression results, three approaches to robustness testing were applied: replacing the core explanatory variables, replacing the response variables, and using the SYS-GMM estimation method. The first approach is to replace the core explanatory variables (Model 3 in Table 3); the per capita GDP is used to replace per capita nighttime light brightness, while a GDP deflator is utilised to adjust nominal GDP by eliminating the effect of price changes. The second is to replace the explained variable with carbon emission intensity (Model 4 in Table 3), which refers to carbon emissions per unit of GDP. In general, a city with a lower carbon emission intensity has a higher level of low-carbon development and is more likely to exhibit decoupling of urban carbon emissions and economic growth. The third approach uses the SYS-GMM estimation method (Model 5 in Table 3). Change in carbon emissions change may have time-dependent path-dependent characteristics (i.e., a time-lag effect) and carbon emissions may exhibit a two-way causal relationship with economic growth, industrial structure, technological progress, and other factors, resulting in the problems of endogeneity. This study therefore referred to Shao et al.’s method [39] and used the SYS-GMM method to verify the inverted U-shaped relationship between carbon emissions and economic growth. This method partially alleviates the problem of endogeneity. Based on the regression results of the robustness test, the coefficient signs and significance levels of the core explanatory variables were consistent with those obtained for in the benchmark regression. This further confirms the inverse U-shaped relationship between carbon emissions and economic growth, indicating that the benchmark regression results are reliable.

**Table 3:** Robustness test regression results.

Variable	*cityemi*	*carbon emission intensity*	*cityemi*
	Model 3	Model 4	Model 5
*GDP* per capita	4.517***		
	(4.55)		
*(GDP* per capita*)*^*2*^	−0.050**		
	(−2.57)		
*L.cityemi*			1.0697***
			(38.57)
*nightlcap*		0.042**	0.0404**
		(2.44)	(2.26)
*nightlcap*^*2*^		−0.0002***	−0.00011**
		(−3.23)	(−2.43)
Control variables	Yes	Yes	Yes
Time-fixed effect	Yes	Yes	Yes
City-fixed effect	Yes	Yes	Yes
Number of id	221	221	221
R-squared	0.338	0.326	–
AR(1)[P]			−3.67[0.00]
AR(2) [P]			2.04[0.04]
AR(3) [P]			−1.00[0.32]
Hansen[p]			113.15[0.232]

Note: ***, **, and * represent the significance at the level of 1 %, 5 %, and 10 %, respectively.

### 3.3 Heterogeneity analysis

#### 3.3.1 Heterogeneity analysis based on low-carbon pilot policies

In 2010, the National Development and Reform Commission of China issued the *Notice on the Pilot Work of Low-carbon Provinces and Cities*. Lists of the second and third batches of low-carbon pilot citites were subsequently released. Currently, 87 provinces and cities have been identified as low-carbon pilot cities. Owing to the lack of available data, some low-carbon pilots were not included in this study. The low-carbon pilot cities included are listed in Table S6. China's low-carbon pilot policy is a constructive step towards promoting green and low-carbon advancement throughout China. This policy aims to transform urban production and lifestyles by utilising low-carbon approaches and technologies, encouraging residents to adopt sustainable consumption practices and driving innovation in clean-process technologies, leading to significant reductions in CO_2_ emissions. Generally, low-carbon pilot cities exhibit a greater decline in carbon intensity per unit GDP than non-pilot cities [40]. Whether low-carbon pilot policies affect carbon emission peaks in cities requires further investigation. Therefore, this study introduces the ‘policy’ dummy variable (*LC*) to evaluate the heterogeneous impact of low-carbon pilot policies (LCPPs) in China. When a prefecture-level city is included in the low-carbon pilot, *LC* = 1, otherwise, *LC* = 0.

The results of the heterogeneity analysis based on low-carbon pilot policies (Table 4) reveal a significant inverted U-shaped curve relationship between city carbon emissions and per capita nighttime light brightness (this curve passed the ‘Utest’ test). Conversely, the coefficients of per capita nighttime light brightness and its quadratic term in the non-low-carbon pilot city group did not achieve statistical significance. Consequently, there was no evidence of a relationship with CKC this population. The results of the heterogeneity analysis conducted on the LCPPs revealed that the implementation of such policies helps prefecture-level cities to achieve peak carbon. This is mainly because these policies compel low-carbon cities to participate more actively in leading and exemplifying structural transformations in industry, optimizing energy structures, transforming lifestyles, and in related processes. For example, most of the second-batch low-carbon pilot cities have set clear targets and roadmaps for achieving peak carbon, leading to positive trends and ongoing efforts to achieve peak carbon. Low-carbon pilot policies have substantially enhanced the understanding of low-carbon development and have built capacity for such cities, consolidating their overall strength and influence.

**Table 4:** Heterogeneity analysis based on low-carbon pilot policies.

Variable	*LC* = 1	*LC* = 0
	cityemi	cityemi
	Model 6	Model 7
*nightlcap*	0.615*	0.121
	(1.93)	(1.31)
*nightlcap*^*2*^	−0.0011**	−0.00024*
	(−2.04)	(−1.75)
Control variables	Yes	Yes
Time-fixed effect	Yes	Yes
City-fixed effect	Yes	Yes
Observations	960	2575
Number of id	60	161
R-squared	0.457	0.247
Utest	Yes	Yes

Note: ***, **, and * represent the significance levels of 1 %, 5 %, and 10 %, respectively. t-statistics are shown in parentheses.

#### 3.3.2 Heterogeneity analysis based on the Hu Line

The Aihui-Tengchong line (Hu Line) is an important demographic and geographical boundary in China, and is a dividing line for disproportionate social and economic development [41,42]. Population density is higher and economic development greater to the east of the line than to the west. Population distribution, economic development, and carbon emissions may differ significantly on either side of the Hu Line [43], highlighting the need to analyse the heterogeneity of the relationship between economic growth and carbon emissions for prefecture-level cities on either side of this line. Therefore, this study introduced a dummy variable *hline* to represent the Hu Line. When a city is located to the east of the Hu Line, *hline* is 1; otherwise, it is 0.

The heterogeneity regression results (Table 5) reveal that the coefficients of per capita nighttime light intensity and its quadratic term were significance test at a 5 % level for east of the Hu Line, but were not significant west of the line. The inverted U-shape curve passes the ‘Utest’ test. This suggests that carbon Kuznets curve applies to prefecture-level cities east, but not west of Hu Line. This heterogeneity in the carbon Kuznets curve results from the significant difference in the overall strength of prefecture-level cities on either side of this line, reflecting disparities in population, economy, investment, energy, urbanisation, and society, with those to the east being significantly stronger than those to the west. As they are constrained by natural conditions, these disparities between the two sides may continue to widen, resulting in further heterogeneity in the carbon Kuznets curve of prefecture-level cities on either side of the line.

**Table 5:** Heterogeneity analysis based on the Hu Line.

Variable	*Hline* = 1	*Hline* = 0
	*cityemi*	*cityemi*
	Model 8	Model 9
*nightlcap*	0.359**	0.420
	(2.55)	(0.88)
*nightlcap*^*2*^	−0.00083**	−0.0011*
	(−2.57)	(−1.77)
Control variables	Yes	Yes
Time-fixed effect	Yes	Yes
City-fixed effect	Yes	Yes
Observations	3312	223
Number of id	207	14
R-squared	0.313	0.614
Utest	Yes	Yes

Note: ***, **, and * represent the significance at the level of 1 %, 5 %, and 10 %, respectively. The t-statistic is shown in parentheses.

#### 3.3.3 Heterogeneity analysis based on city population size

Urban population size is a key criterion for measuring the size of a city, and the size of a city in China is mainly categorized according to its permanent population. Cities of different levels and sizes differ in economic growth, natural resource endowment, industrial structure, and technological level, leading to differences in carbon emissions [44]. To explore the inverse U-shaped relationship between carbon emissions and economic growth in cities of different sizes, this study divided city sizes into three levels according to city population—large-scale cities (population >10 million), medium-scale cities (3 million to 10 million), and small-scale cities (<3 million)—according to the *Notice on Adjusting the scale delineation Standards* (The State Council of China) and referring to the method of Weng et al. [44].

The regression results (Table 6) reveal a significant inverted U-shaped relationship between carbon emissions and economic growth in medium-scale cities (passing the Utest); no such relationship was detected for large- and small-scale cities. This may be because medium-scale cities, concentrated primarily in the central and eastern regions, exhibit rapid economic development and focus on improving environmental technology and resource utilisation efficiency and adjusting and upgrading of industrial structure. This also suggests that it is appropriate to keep the city population size at the medium scale, as no significant inverted U-shaped relationship was observed or small- and large-scale cities.

**Table 6:** Heterogeneity analysis based on city size.

variable	Larger scale	Medium scale	Small scale
	*cityemi*	*cityemi*	*cityemi*
	Model10	Model11	Model12
*nightlcap*	−2.455	0.731**	0.149
	(−0.69)	(2.15)	(1.41)
*nightlcap*^*2*^	0.071	−0.004**	−0.000
	(1.24)	(−2.13)	(−1.65)
Control variables	Yes	Yes	Yes
Time-fixed effect	Yes	Yes	Yes
City-fixed effect	Yes	Yes	Yes
Observations	166	2204	1165
Number of id	15	148	78
R-squared	0.498	0.263	0.318
Utest	No	Yes	Yes

Note: ***, **, and * represent the significance at the level of 1 %, 5 %, and 10 %, respectively. t-statistics are shown in parentheses.

## 4 Discussion

The validity of the CKC remains unclear, owing to the substantial differences in the published findings, with some findings supporting it and others contradicting it. Studies that do not support the CKC propose linear, positive U-shaped, N-shaped, or inverted N-shaped relationships [[45], [46], [47], [48]]. The current findings are consistent with the CKC. The benchmark regression results significantly support the CKC. Robustness testing using three different approaches supported the benchmark regression results. The prefecture-level city panel data used here are more persuasive than provincial panel or time-series data. Nighttime brightness was used as an indicator of economic growth. Nighttime brightness is a good alternative indicator of GDP [25,26]. For instance, Chen et al. used nighttime brightness to estimate CO_2_ emissions [49,50] and assumed that endogeneity between CO_2_ emissions and nighttime brightness cannot be avoided. Therefore, this study used the SYS-GMM estimation method to alleviate endogeneity issues.

CKC is not a new topic, and its existence in China has been widely studied. Nonetheless, few studies have estimated the inflection point in the CKC or considered per capita GDP data; this study addresses this gap. China requires per capita GDP growth of at least 6.6 % to achieve peak carbon before 2030. If its population size remained constant, its GDP growth rate would reach 6.6 %. Before 2030, China's GDP growth is predicted to reach 4.0 % [51,52] or higher [6], with few studies predicting that it will exceed 6.5 %. It will therefore be difficult for China to achieve peak carbon by 2030 under current conditions.

## 5 Conclusion and policy implications

Nighttime light data of prefecture-level cities in China from 2003 to 2018 were analysed using a two-way fixed effect model to examine the existence of an inverted U-shaped relationship between carbon emissions and economic growth. The key findings are as follows: (1) There is a significant inverted U-shaped relationship between per capita nighttime light brightness and carbon emissions, supporting the CKC hypothesis. The robustness test confirms this conclusion. And considering that the GDP per capita is still lower than 139.8 thousand Yuan corresponding to the turning point calculating by this research, it means that the CO_2_ emission would be still increasing in the near future. And it is necessary to strive for an economic growth rate of 6 per cent or more to reach to the critical point of the GDP per capita as early as possible. (2) Cities that have implemented low-carbon pilot policies may reach their carbon peaks earlier. In prefecture-level cities that have implemented low-carbon pilot policies, there is a significant inverted U-shaped relationship between carbon emissions and per capita nighttime light brightness, however no significant inverted U-shaped relationship were observed for cities that have not implemented low-carbon pilot policies. (3) The peak carbon is expected to occur earlier in eastern China than in western China. Specifically, an inverted U-shaped relationship exists between urban carbon emissions and economic growth to the east, but not to the west, of the Hu Line.

Based on these research findings, the following measures can be implemented to achieve peak carbon: First, cities should adhere to high-quality economic growth and accelerate the transformation of economic growth modes. It is essential to construct a locally tailored modern economic and industrial system based on the specific characteristics of prefecture-level cities. China should implement new development concepts, foster deep integration between local development and urban agglomerations, and promote regional economic integration and coordinated development. Second, LCPPs should be improved and should be implemented in more cities. Based on evaluations of the effectiveness of current LCPPs, the roll-out of these pilot projects will be expanded to over 100 cities. Each pilot city should explore low-carbon and green development models and paths appropriate for its region. Third, efforts to achieve peak carbon in the eastern cities should be accelerated and prioritised over such efforts in western cities. Considering that most Chinese cities have not yet achieved peak carbon, cities in every region must implement the “*Action Plan for Peaking Carbon Emissions Before 2030*”, and evaluate their local economic and social development realities, natural resource, and environmental factors to systematically achieve peak carbon in a sequence from east to west.

In terms of its limitations, this paper omitted prefecture-level cities lacking sufficient data to establish a balanced panel, leading to information loss. This research collected panel data only for the period 2003–2018 because other years could not be accessed using the data currently available.

## Data availability statement

Data or code for this study are available from the first author upon reasonable request.

## Funding

This study was supported by the 10.13039/501100001809National Natural Science Foundation of China (72104112; 41901255), and the Development Special Program of Jiangsu Provincial Federation of Philosophy and Social Sciences (23GSA-012).

## CRediT authorship contribution statement

**Xiaoqi Zheng:** Writing – original draft, Methodology, Data curation, Conceptualization. **Jiaying Wang:** Writing – original draft, Data curation. **Xiangbo Xu:** Writing – review & editing, Writing – original draft, Validation, Methodology, Conceptualization. **Ran Yu:** Methodology, Conceptualization. **Sheng Zhang:** Methodology, Conceptualization.

## Declaration of competing interest

The authors declare that they have no known competing financial interests or personal relationships that could have appeared to influence the work reported in this paper.
