# Supplementary material for: Carbon Kuznets curve in China: Nighttime light analysis in prefecture-level cities
Source: Heliyon. 2024 Aug 14;10(16):e36312. doi: 10.1016/j.heliyon.2024.e36312 (PMC11382192; doi:10.1016/j.heliyon.2024.e36312)
Supplement: Multimedia component 1 [file mmc1.docx]

Carbon Kuznets Curve in China: Nighttime Light Analysis in Prefecture-level Cities

Xiaoqi Zheng ^a^, Jiaying Wang ^a^, Xiangbo Xu^b*^, Ran Yu^c^, Sheng Zhang^d^

*^a^* School of Economics, Nanjing University of Posts and Telecommunications, No.9, Wenyuan Road, Nanjing, 210023, China, zhengxiaoqi@njupt.edu.cn (Xiaoqi Zheng), wangjiaying1219@163.com (Jiaying Wang)

*^b^* Institute of Geographical Science and Resources, Chinese Academy of Sciences, 11A, Datun Road, Chaoyang District, Beijing, 100101, China, ydxu.ccap@igsnrr.ac.cn

*^c^* School of Environment, Renmin University of China, No. 59 Zhongguancun Street, Haidian District Beijing, 100872, China, yuran19950502@ruc.edu.cn

*^d^* School of Environment, Tsinghua University, No. 30 Shuangqing Road, Haidian District Beijing, 100084, China, zhangsheng0531@tsinghua.edu.cn

**Table S1** The results of correlation coefficient Test

| variable | *nightlcap* | *lpopden* | *lcaplar* | *indupd* | *urban* | *ltotele* |
| --- | --- | --- | --- | --- | --- | --- |
| *nightlcap* | 1 |  |  |  |  |  |
| *lpopden* | 0.156*** | 1 |  |  |  |  |
| *lcaplar* | 0.045*** | 0.047*** | 1 |  |  |  |
| *indupd* | -0.053*** | -0.075*** | -0.065*** | 1 |  |  |
| *urban* | 0.495*** | 0.128*** | 0.271*** | 0.219*** | 1 |  |
| *ltotele* | 0.274*** | 0.427*** | 0.288*** | 0.123*** | 0.626*** | 1 |

**Table S2** The result of unit root test under LLC test or IPS test

| Variable | Adjusted t | p-value |
| --- | --- | --- |
| *cityemi* | -13.7988 | 0.0000 |
| *nightlcap* | -22.2219 | 0.0000 |
| *nightlcap2* | -15.8226 | 0.0000 |
| *lpopden* | -36.8704 | 0.0000 |
| *lcaplar* | -16.7811 | 0.0000 |
| *indupd* | -5.0983 | 0.0000 |
| *urban* | -81.1027 | 0.0000 |
| Variable | W-t-bar | p-value |
| *ltotele* | -14.2490 | 0.0000 |

**Table S3** The results of co-integration test

|  | Westerlund test | p-value |
| --- | --- | --- |
| Variance ratio | 6.0762 | 0.0000 |

**Table S4** The Hausman test

| variable | fe | re |
| --- | --- | --- |
|  | *cityemi* | *cityemi* |
| *nightlcap* | 0.298*** | 0.131*** |
|  | (6.00) | (3.12) |
| *nightlcap2* | -0.001*** | -0.0003*** |
|  | (-5.42) | (-3.40) |
| *lpopden* | 5.686 | -3.034 |
|  | (0.99) | (-1.53) |
| *lcaplar* | -0.638 | -0.022 |
|  | (-1.16) | (-0.04) |
| *indupd* | 1.170 | 1.750 |
|  | (0.94) | (1.47) |
| *urban* | 23.973*** | 21.601*** |
|  | (6.01) | (5.54) |
| *ltotele* | 13.005*** | 14.180*** |
|  | (15.86) | (18.52) |
| Constant | -177.139*** | -144.666*** |
|  | (-5.63) | (-11.69) |
| Observations | 3,535 | 3,535 |
| Number of id | 221 | 221 |
| Huasman test | Prob>chi2 = 0.0000 | |

Note: ***, ** and * represent the significance at the level of 1%, 5% and 10%, respectively. The t-statistic is shown in parentheses.

**Table S5** The corresponding relationship between per capita GDP and per capita nighttime light brightness

| variable | per capita GDP |
| --- | --- |
| *nightlcap* | 0.042*** |
|  | (12.70) |
| Constant | 2.879*** |
|  | (41.55) |
| Observations | 3,536 |
| R-squared | 0.222 |

Note: ***, ** and * represent the significance at the level of 1%, 5% and 10%, respectively. The t-statistic is shown in parentheses.

**Table S6** The low-carbon pilot cities that were included in this study

| First batch | Second batch | Third batch |
| --- | --- | --- |
| Shanghai | Beijing | Wuhai |
| Yinchuan | Shanghai | Shenyang |
| Nanjing | Shijiazhuang | Dalian |
| Changzhou | Qinhuangdao | Zhaoyang |
| Suzhou | Jincheng | Nanjing |
| Guangzhou | Hulunbeier | Changzhou |
| Xiamen | Suzhou | Jiaxing |
| Shenyang | Huai’an | Jinhua |
| Chongqing | Zhenjiang | Quzhou |
| Baoding | Ningbo | Hefei |
| Guiyang | Wenzhou | Huaibei |
| Shenzhen | Chizhou | Huangshan |
| Nanchang | Nanping | Lu’an |
| Hangzhou | Jingdezhen | Xuancheng |
|  | Ganzhou | Sanming |
|  | Qingdao | Ji’an |
|  | Wuhan | Jinan |
|  | Guangzhou | Yantai |
|  | Guilin | Weifang |
|  | Guangyuan | Changsha |
|  | Zunyi | Zhuzhou |
|  | Kunming | Xiangtan |
|  | Yan’an | Chenzhou |
|  | Wulumuqi | Zhongshan |
|  |  | Liuzhou |
|  |  | Chengdu |
|  |  | Lanzhou |
|  |  | Yinchuan |
